# Impaired immune tolerance mediated by reduced Tfr cells in rheumatoid arthritis linked to gut microbiota dysbiosis and altered metabolites

**DOI:** 10.1186/s13075-023-03260-y

**Published:** 2024-01-13

**Authors:** Ruihe Wu, Dongming Wang, Liyun Cheng, Rui Su, Baochen Li, Chunxue Fan, Chong Gao, Caihong Wang

**Affiliations:** 1 Department of Rheumatology, The Second Hospital of Shanxi Medical University, Taiyuan, Shanxi China; 2 Shanxi Key Laboratory of Immunomicroecology, Taiyuan, Shanxi China; 3 Department of Orthopedics, The Second Hospital of Shanxi Medical University, Taiyuan, China; 4 Department of Pathology, Brigham and Women’s Hospital, Harvard Medical School, Boston, MA USA

**Keywords:** Rheumatoid arthritis, Gut-joint axis, Gut microbiota, Metabolites, Follicular regulatory T cells, Immune tolerance

## Abstract

**Background:**

Patients with rheumatoid arthritis (RA) showed impaired immune tolerance characterized by reduced follicular regulatory T (Tfr) cells, and they also exhibited altered gut microbiotas and their metabolites in RA. However, the association of gut microbiotas and their metabolites with the immune tolerance mediated by Tfr cells in RA remains unclear.

**Methods:**

Peripheral blood and stool samples were collected from 32 new-onset RA patients and 17 healthy controls (HCs) in the Second Hospital of Shanxi Medical University between January 2022 and June 2022. The peripheral blood was used to detect the circulating regulatory T (Treg), helper T(Th)17, Tfr, and follicular helper T (Tfh) cells by modified flow cytometry. The stool samples were used to analyze the gut microbiotas and their metabolites via 16S rDNA sequencing and metabolomic profiling. We aimed to characterize the gut microbiotas and their metabolites in RA and identified their association with Tfr cell-mediated immune tolerance.

**Results:**

The new-onset RA demonstrated reduced Treg and Tfr cells, associated with the disease activity and autoantibodies. There were significant differences in gut microbiotas between the two groups as the results of β diversity analysis (*P* = 0.039) including 21 differential gut microbiotas from the phylum to genus levels. In which, *Ruminococcus 2* was associated with the disease activity and autoantibodies of RA, and it was identified as the potential biomarker of RA [area under curve (AUC) = 0.782, 95% confidence interval (CI) = 0.636–0.929,* P* = 0.001]. Eleven differential metabolites were identified and participated in four main pathways related to RA. Arachidonic acid might be the potential biomarker of RA (AUC = 0.724, 95% CI = 0.595–0.909, *P* = 0.038), and it was the core metabolite as the positive association with six gut microbiotas enriched in RA. The reduced Tfr cells were associated with the altered gut microbiotas and their metabolites including the *Ruminococcus 2*, the arachidonic acid involved in the biosynthesis of unsaturated fatty acid pathway and the 3-methyldioxyindole involved in the tryptophan metabolism pathway.

**Conclusion:**

The breakdown of immune tolerance mediated by reduced Tfr cells was associated with the altered gut microbiotas and their metabolites implying the possible mechanism of RA pathogenesis from the perspective of microecology-metabolism-immune.

**Supplementary Information:**

The online version contains supplementary material available at 10.1186/s13075-023-03260-y.

## Background

Rheumatoid arthritis (RA) is a highly disabling autoimmune disease characterized by persistent synovitis and joint damage [1, 2]. Excessive production of abnormal autoantibodies, including anti-cyclic peptide containing citrulline (anti-CCP), play a pivotal role in the onset and progression of RA, which can even be detected in the stage of pre-clinical RA (Pre-RA) [3, 4]. A comprehensive exploration of the intricate process of antibody generation is vital to achieving targeted therapy, early remission, and the prevention of RA. The complex etiopathogenesis of RA involves the loss of immune tolerance resulting from genetic and environmental factors, which has been confirmed to be a crucial contributor to the over-production of abnormal antibodies, and it always occurs in the Pre-RA stage to promote the further progression of RA [5, 6]. Therefore, inducing and restoring immune tolerance are promising in the prevention and treatment of RA.

The CD4 + CD25 + Forkhead Box 3 (FoxP3) + regulatory T (Treg) cells play a critical role in maintaining immune tolerance not only by producing the anti-inflammatory cytokine (such as interleukin-10) but also by suppressing the activation and proliferation of effector T cells, specifically helper T(Th)17 cells, which secret the pro-inflammatory cytokine, interleukin-17 [7]. It has been reported that RA patients had an imbalance of Th17/Treg cells, especially the reduced Treg cells [8, 9]. Exploiting the suppressive capacities of Treg cells to enhance immune tolerance has been an emerging field to treat autoimmune diseases such as the application of low-dose interleukin-2 (IL-2) in RA [10, 11]. However, the breakdown of immune tolerance mediated by the aberrant Treg cells seems to be difficult to explain the over-production of antibodies in RA.

The autoantibody production in RA depends on the response of lymphoid follicular germinal centers (GCs), which are essential for B cells to complete the series of reactions including affinity maturation, class switch recombination, and somatic hypermutation to produce a large number of high-affinity antibodies and memory B cells finally [12]. The discovery of a novel CD4 + T subset cells localized in lymphoid follicular GCs is termed follicular regulatory T (Tfr) cells, which is characterized by the expression of C-X-C chemokine receptor type 5 (CXCR5, a chemokine receptor homing to the T-cell zone) and Foxp3 [13–15]. While the exact differentiation mechanism of Tfr cells is still poorly defined, existing evidence supports that they originate de novo from thymic-derived FoxP3 + Treg precursors under multiple stimulations. As a specific subpopulation of Treg cells, Tfr cells contribute to maintaining immune tolerance by inhibiting follicular helper T (Tfh) cells, another GC-residing cell type to facilitate the production of antibodies by promoting the formation and response of GC [16, 17]. The function of Tfr cells provides a new understanding of maintaining immune tolerance and antibody production, which may contribute to the further exploration of RA pathogenesis. It has been found that RA patients had imbalanced Tfr/Tfh cells [18, 19], especially those with decreased Tfr cells exhibited high disease activity and antibodies, suggesting that the aberrant Tfr cells could lead to the over-production of antibodies to destroy the immune tolerance. And those with active RA showed higher Tfh cells which was associated with the enhanced IL-6/pSTAT3 signaling [20]. Of note, the imbalance was altered after treatment and patients with RA in stable remission with lower levels of autoantibodies exhibited increased Tfr cells [21, 22], indicating that targeting Tfr cells to restore immune tolerance had significant therapeutic potential for RA. It has become a consensus that impaired immune tolerance and antibody production are the core pathogenesis of RA and the Pre-RA, and Tfr cells play an important role in inhibiting the production of antibodies and maintaining immune tolerance. Therefore, the impaired immune tolerance and the over-production of antibodies caused by aberrant Tfr cells play a crucial role in the pathogenesis of RA. Nevertheless, the potential upstream factors regulating Tfr cell-mediated immune tolerance remain to be fully elucidated, which is the aim of our study.

There is substantial evidence suggesting that the initiation of RA might originate in mucosal sites far away from joints, such as the gut, which emphasizes the effect of the gut-joint axis exerted in RA [23, 24]. The gut microbiota as the most critical component in the gut is important for balancing health and disease, and it is associated with autoimmune diseases including RA [25, 26]. Some significant studies have reported that gut microbiota dysbiosis in RA was correlated with disease activity [27–29]. For instance, patients with RA exhibited elevated *Prevotella copri*, particularly those with high disease activity [27, 28], while the reduced *Haemophilus* spp. in untreated RA patients was associated with high-level abnormal autoantibodies negatively [29]. Gut microbiota plays an immunomodulatory role in maintaining immune homeostasis under normal conditions [30], while gut microbiota dysbiosis could activate both innate and adaptive immune cells, which may serve as the mechanistic connection between mucosal changes and arthritis development [31, 32]. Moreover, gut microbiota-derived metabolites including short-chain fatty acids (SCFAs), bile acids and tryptophan and its derivatives have been recognized as the crosstalk of the gut-joint axis to exert effects [33, 34]. Altered metabolite profiles have been observed in RA patients indicating their significant role in the pathological mechanism of RA [35, 36]. The main mechanism driving the onset of RA through the gut-joint axis revolves around the impact of gut microbiotas and their metabolites to activate pro-inflammatory immune cells and promote their trafficking from the gut to joints [37, 38]. Notably, the altered gut microbiotas and their metabolites may also play a role in the gut-joint axis by destroying immune tolerance. The present studies focus on whether and how gut microbiotas and their metabolites affect immune tolerance mediated by Treg and Tfr cells to exert critical components within the gut-joint axis. To date, some studies on collagen-induced arthritis (CIA) and SKG arthritis models have found that microbiota-derived butyrate might suppress autoantibody production and ameliorate arthritis by enhancing the Treg and Tfr cells [39, 40]. However, there are numerous gut microbiotas and their metabolites in RA, whether other gut microbiotas and metabolites are involved in the pathogenesis of RA, and how their relationship with Treg cells, especially Tfr cells, still needs further systematic research. Thus, it is necessary to find the disease biomarkers of RA from the numerous gut microbiotas and their metabolites and to analyze their relationship with Treg and Tfr cells, which is the focus of our study.

Therefore, we performed the study to explore the association of gut microbiotas and their metabolites with the immune tolerance mediated by Tfr cells in RA. First, we assessed the immune tolerance status in RA by detecting the expression of Th17, Treg, Tfr, and Tfh cells in the peripheral blood via modified flow cytometry. And then, considering that the detection of gut microbiota and metabolites in stool samples is an ideal method to study the direct correlation between gut microbiotas and their metabolites, we identified the characteristics of gut microbiotas and their metabolites in RA by the combination of 16S rDNA sequencing and ultra-performance liquid chromatography-tandem mass spectrometry (UPLC-MS)-based untargeted metabolomic profiling. Subsequently, we explored the association of gut microbiotas and their metabolites with the immune tolerance mediated by circulating Tfr cells. The results of our study aimed to provide a novel insight into the pathogenesis of RA from the perspective of gut microbiota-metabolite-immune tolerance.

## Materials and methods

### Study population and data

This study recruited new-onset RA patients who were admitted to the Second Hospital of Shanxi Medical University between January 2022 and June 2022. Participants should meet the criteria of the 2010 ACR/EULAR classification and diagnosis criteria for RA and should not receive any steroid, disease-modifying antirheumatic drugs (DMARDs), or biological agents for at least 3 months. Healthy volunteers with no history of autoimmune diseases or abnormal clinical indicators were included as healthy controls (HCs). Several exclusion criteria were applied to both the new-onset RA patients and HCs as follows: (i) recent use of antibiotics and microecological agents within 8 weeks; (ii) suffered from malignant tumors, severe infections, or serious cardiovascular system diseases; (iii) suffered from inflammatory bowel disease and other diseases that may seriously affect the gut microbiotas and their metabolites; (iv) a history of gastrointestinal surgery. In accordance with these criteria, the final cohort comprised a total of 32 new-onset RA patients and 17 HCs. This study was carried out under the principles outlined in the Declaration of Helsinki and received approval from the Ethics Committee of the Second Hospital of Shanxi Medical University (Approval (2021) YX No. (250)). All participants provided written informed consent.

Stool samples from HCs and new-onset patients with RA were collected and preserved at a temperature of − 80 °C for subsequent processing. Blood samples from each participant were also processed upon collection to determine the expression of Th17, Treg, Tfr, and Tfh cells in peripheral blood. Additionally, demographic data, clinical data [including the tenderness joint count (TJC), swollen joint count (SJC), and disease activity score 28(DAS28)] as well as laboratory tests [including erythrocyte sedimentation rate (ESR, mm/h), anti-CCP (U/ml), rheumatoid factor (RF)-Ig M(U/ml) and RF-Ig G(U/ml)] were acquired for analysis.

### The detection of Th17, Treg, Tfr and Tfh cells

Peripheral blood samples were collected in heparin anticoagulation tubes for the assessment of circulating Th17, Treg, Tfr, and Tfh cells by modified flow cytometry. A representative flow cytometry figure is illustrated in Additional file 1: Fig. S1. The specific details of the experimental procedures were in the “Methods” section of Additional file 1.

### The analysis of gut microbiota and fecal metabolite

Considering the susceptibility of gut microbiotas and their metabolites to diverse influences such as medication and dietary habits, meticulous consideration was given to mitigate potential biases. Therefore, participants were all from Shanxi Province to ensure dietary comparability and refrained from medication for at least 8 weeks before sample collection. The gut microbiotas and their metabolites were assessed by the combination of 16S rDNA sequencing and UPLC-MS-based untargeted metabolomic profiling. Further details were in the “Methods” section of Additional file 1.

### Statistical analysis

The analysis of the demographic data, clinical data, and laboratory tests was under IBM SPSS 25.0. The normally distributed continuous variables were presented as mean ± standard deviation (SD) and analyzed by the independent samples *t*-test. While the nonparametric variables were presented as median (Q1, Q3) and were analyzed by the Mann–Whitney *U* test. Categorical variables were described by ratio or percentage and were assessed by the chi-square (*χ*^2^) or Fisher’s exact tests. All *P*-values were two-tailed and statistical significance was defined as *P* < 0.05.

## Results

### Characteristics of participants

There were 23 females and 9 males among the 32 new-onset RA patients with an average age of 56.78 ± 11.69 years. And 13 females and 4 males were included in 17 HCs with an average age of 51.94 ± 13.03 years. No significant disparities were observed in terms of gender (*P* = 0.729) or age (*P* = 0.735) between the new-onset RA patients and the HCs. The summary of demographic data, clinical data, and laboratory tests of new-onset RA patients and HCs is presented in Table 1.

**Table 1:** The summary of demographic data, clinical data and laboratory tests

	HC (*n* = 17)	New-onset RA (*n* = 32)	*P* value
**Demographic data**			
Age (years)^a^	51.94 ± 13.03	56.78 ± 11.69	0.753
Sex(male/female)^b^	4/13	9/23	0.729
**Clinical data**			
Course(months)^c^	-	12(3,57)	-
TJC^c^	-	9(4,20)	-
SJC^c^	-	6(2,18)	-
DAS28^c^	-	5.38(4.45,6.97)	-
**Laboratory tests**			
ESR (mm/h)^c^	-	58.50(31.00,84.50)	-
Anti-CCP (U/ml)^c^	-	124.37(56.71,223.37)	-
RF-IgM (U/ml)^c^	-	288.10(59.25,300.00)	-
RF-IgG (U/ml)^c^	-	106.30(38.2,196.20)	-

*Abbreviations*: *HC* healthy control, *RA* rheumatoid arthritis, *TJC* tenderness joint count, *SJC* swollen joint count, *DAS 28* Disease Activity Score 28, *ESR* erythrocyte sedimentation rate, *anti-CCP* anti-cyclic peptide containing citrulline, *RF* rheumatoid factor

^a^Results were expressed as the mean ± SD and were analyzed by independent samples *t*-tests

^b^Categorical variables were described as rates and percentages and were assessed using the chi-square or Fisher’s exact tests

^c^Results were expressed as the median (Q1, Q3) and were analyzed by Mann–Whitney *U* test

### Reduced Treg and Tfr cells in RA were associated with the disease

**Fig. 1:**
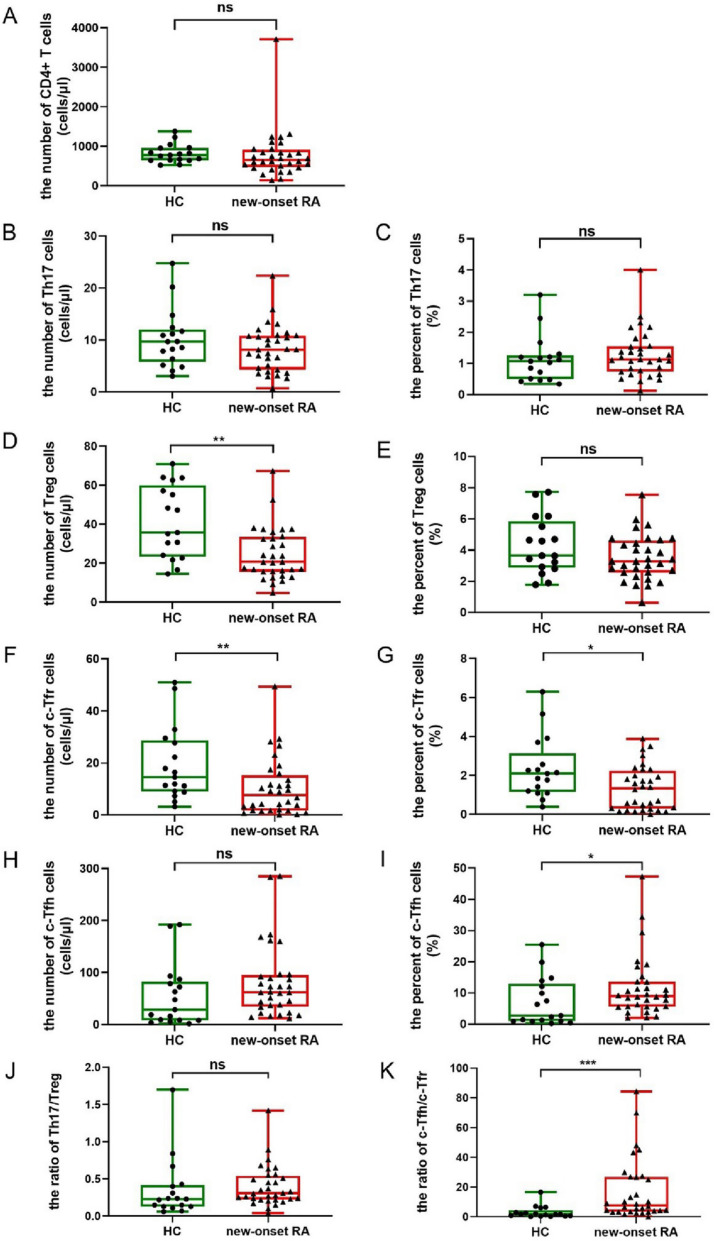
The comparisons in the expressions of CD4 + T, Th17, Treg, c-Tfr, and c-Tfh cells between patients with RA and HCs. The number of Th17 and c-Tfh cells as well as the ratio of Th17/Treg cells was not changed between RA and HCs, while the number of Treg and c-Tfr cells was reduced in new-onset RA patients. And the ratio of c-Tfh/c-Tfr cells was increased in new-onset RA patients. The new-onset patients with RA had a higher percentage of c-Tfh cells but a lower percentage of c-Tfr cells than those in HCs. **A** The comparison in the number of CD4+ T cells. **B** The comparison in the number of Th17 cells. **C** The comparison in the percent of Th17 cells. **D** The comparison in the number of Treg cells. **E** The comparison in the percent of Treg cells. **F** The comparison in the number of c-Tfr cells. **G** The comparison in the percent of c-Tfr cells. **H** The comparison in the number of c-Tfh cells. **I** The comparison in the percent of c-Tfh cells. **J** The comparison in the ratio of Th17/Treg. **K** The comparison in the ratio of c-Tfh/c-Tfr. (Th17: helper T 17 cells; Treg: regulatory T cells; Tfr: follicular regulatory T cells; Tfh: follicular helper T cells; RA: rheumatoid arthritis; HCs: healthy controls)

The comparison of circulating Th17, Treg, Tfr, and Tfh cells between RA and HCs is summarized in Fig. 1 and Additional file 1: Table S1.

Compared with HCs, new-onset RA patients had a lower number of Treg cells [20.605(15.633,33.415) cells/µl *vs.* 35.700(23.360,59.855) cells/µl, *P* = 0.004] and a lower number of c-Tfr cells [7.690(1.700,15.202) cells/µl *vs.* 14.519 (8.979,28.602) cells/µl, *P* = 0.008] resulting in a higher ratio of c-Tfh/c-Tfr [7.822(3.546,26.824) *vs.* 1.916(0.684,4.483), *P* < 0.001], and they also had a lower percentage of c-Tfr cells [1.332(0.282,2.224) % *vs.* 2.097(1.143,3.130) %, *P* = 0.032] but a higher percentage of c-Tfh cells [8.955 (5.580,13.550) % *vs.* 2.750(0.923,12.965) %, *P* = 0.044]. Considering that most Tfr cells are derived from thymic-derived FoxP3 + Treg cells (natural Treg, nTreg) [13–15], our study analyzed the correlation between the number of Treg cells and Tfr cells in the new-onset RA patients, and the results showed that the two were correlated positively, supporting that there was a direct relationship between the two cells in RA (Additional file 1: Fig. S2).

**Fig. 2:**
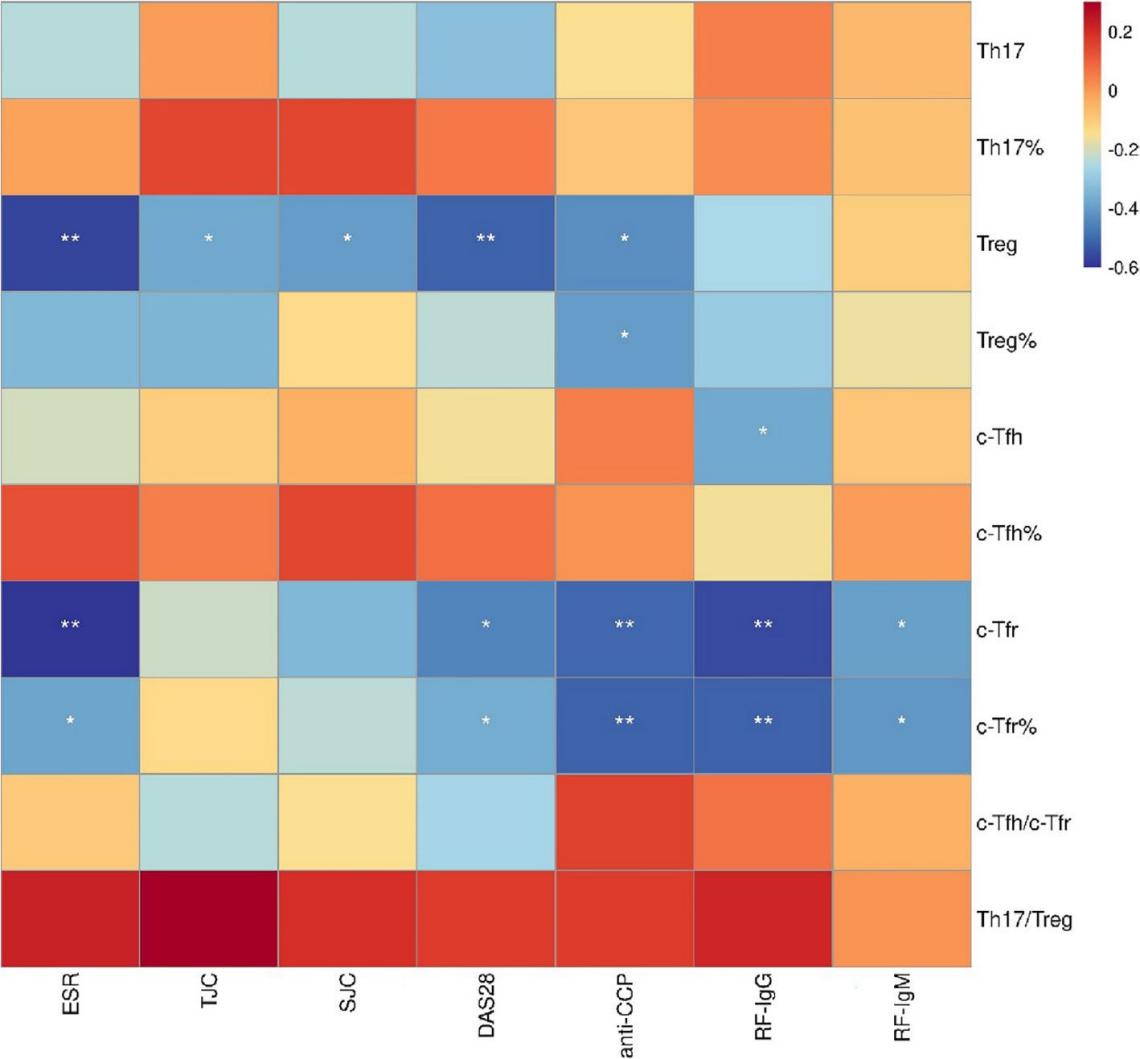
The correlation heatmap of the expression of Th17, Treg, Tfr, and Tfh cells with the clinical indicators of RA. (Th17: helper T 17 cells; Treg: regulatory T cells; Tfr: follicular regulatory T cells; Tfh: follicular helper T cells; RA: rheumatoid arthritis; HCs: healthy controls; ESR: erythrocyte sedimentation rate; TJC: tenderness joint count; SJC: swollen joint count; DAS28: disease activity score 28; anti-CCP: anti-cyclic peptide containing citrulline; RF: rheumatoid factor)

The correlation heatmap of Th17, Treg, Tfr, and Tfh cells with the clinical indicators of RA was conducted (Fig. 2). The number of Treg cells was related to ESR, TJC, SJC, DAS28, and anti-CCP negatively, while the percentage of Treg cells was only related to anti-CCP negatively. The level of c-Tfr cells was negatively associated with ESR, DAS28, anti-CCP, RF-IgG, and RF-IgM. The number of c-Tfh cells was negatively associated with RF-IgG. In summary, the reduced Treg and Tfr cells in RA were associated with the disease activity and the production of autoantibodies of RA.

### Relationship between gut microbiota and RA

**Fig. 3:**
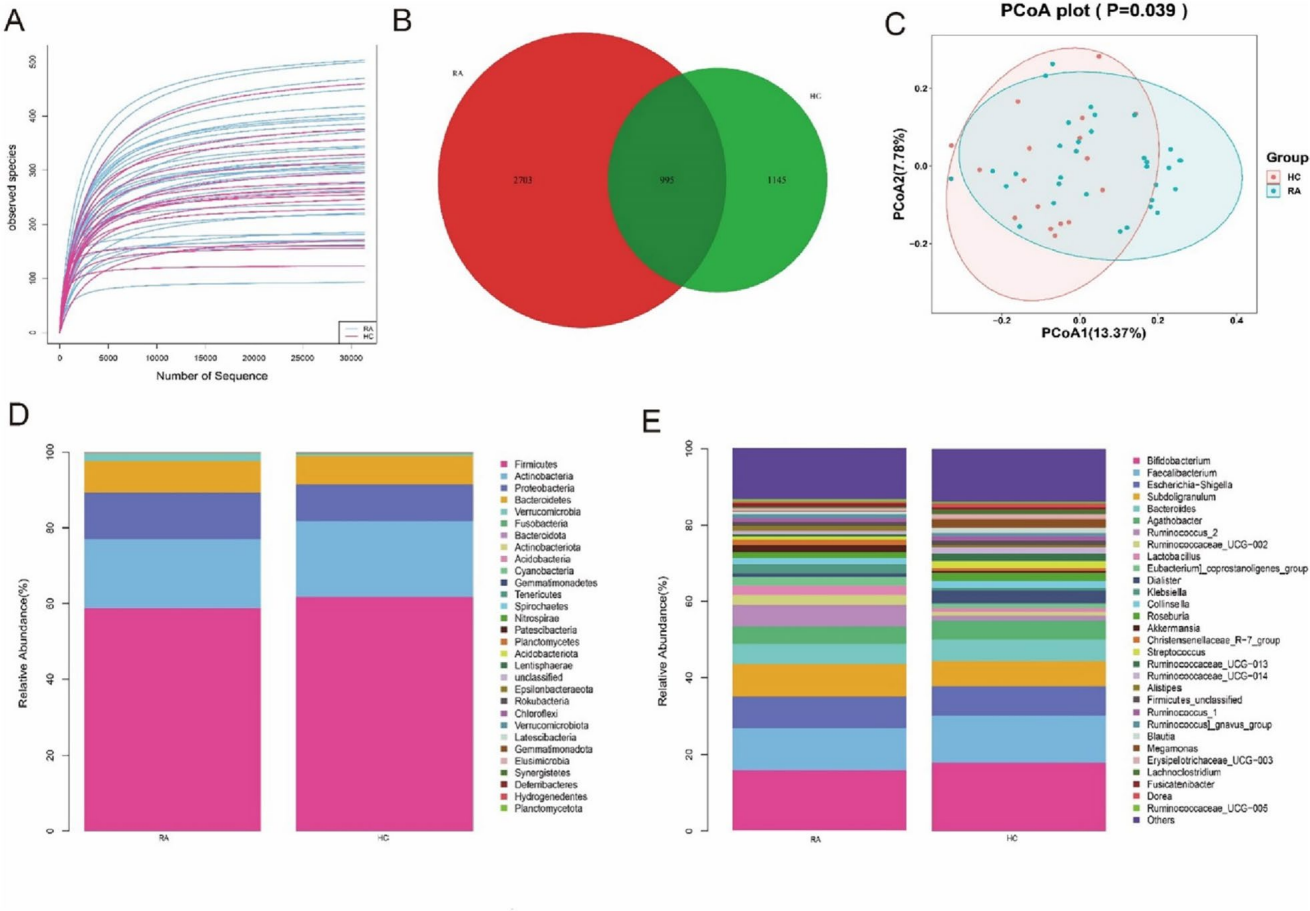
The overview of gut microbiota in new-onset RA patients and HCs. **A** The sequencing depth of the gut microbiota. The curve of each sample was nearly smooth, indicating that there was sufficient sequencing information from these samples in the RA and HCs. **B** The number of feature data in RA and HCs. **C** The β diversity analysis evaluated by principal coordinate analysis (PCoA) score showed that the composition of gut microbiota was significantly different between RA and HCs. **D** The relative abundance of gut microbiota at the phylum level in RA and HCs. **E** The relative abundance of gut microbiota at the genus level in RA and HCs. (RA: rheumatoid arthritis; HCs: healthy controls)

The adequacy of sequencing information from gut microbiota profiles was confirmed by the rarefaction curve based on observed species (Fig. 3A). There were 3698 and 2140 feature data obtained by the DADA2 algorithm from the new-onset RA patients and HCs, and 995 feature data were shared by the new-onset RA patients and HCs (Fig. 3B). Analysis of α diversity indicated that species richness and evenness of gut microbiota were similar between new-onset RA patients and HCs (Additional file 1: Fig. S3). Meanwhile, the β diversity analysis evaluated by the principal coordinate analysis (PCoA) score and the *P* value obtained from the analysis of similarities (ANOSIM) (R = 0.104,* P* = 0.039) showed the composition of gut microbiota had significant differences between new-onset RA patients and HCs (Fig. 3C).

Given that many identified gut microbiotas were not classified at the species level, we mainly focused on the genus level. We compared the differences in the composition of gut microbiota among the top 30 at the phylum and genus level between RA and HCs (Fig. 3D, E). There were three significantly different gut microbiotas at the phylum level and seven gut microbiotas at the genus level (Additional file 1: Table S2).

**Fig. 4:**
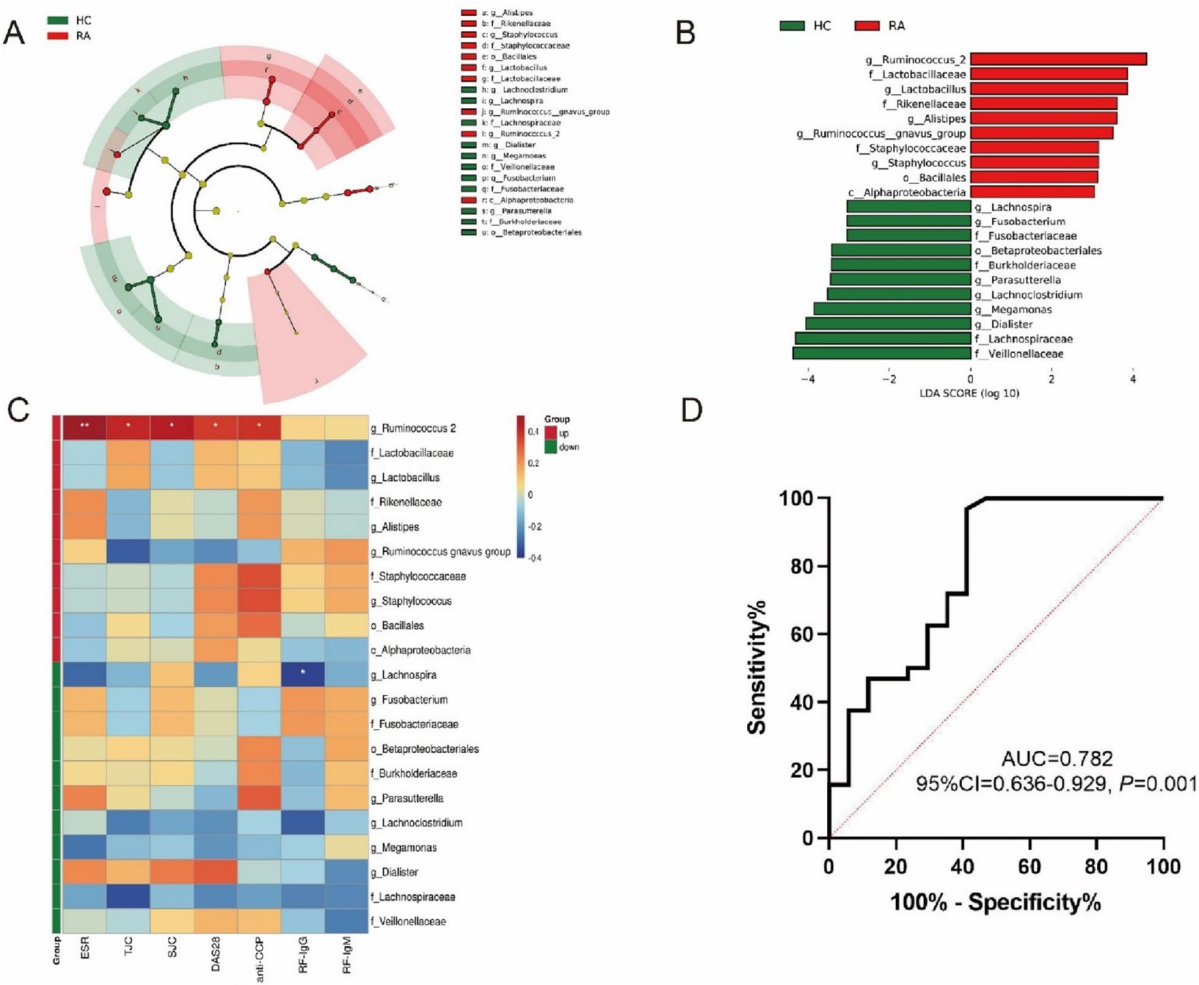
The identification of the differentially abundant gut microbiota in RA and the relationship of the gut microbiota dysbiosis with RA. **A** The phylogenetic distribution in cladogram of differential gut microbiota between RA and HCs. **B** The differential gut microbiota with LDA score > 4 and *P* < 0.05 between RA and HCs, and *Ruminococcus 2* was the differential gut microbiota with the highest LDA score at the genus level in RA. **C** The correlation heatmap of the differentially abundant gut microbiota and the disease activity of RA. **D** The ROC curve of biomarker analysis for *Ruminococcus 2.* (The letters c, o, f and g represent class, order, family, and genus, respectively. RA: rheumatoid arthritis; HCs: healthy controls; LDA: linear discriminant analysis; ROC: receiver operating characteristic)

A total of 21 gut microbiotas from phylum to genus levels were recognized as differential gut microbiota between the RA and HCs by linear discriminant analysis (LDA) effect size (LDA > 3, *P* < 0.05) (Fig. 4A, B). The correlation heatmap of the 21 differential gut microbiotas and the clinical indicators of RA revealed that the upregulated gut microbiota in RA such as *Ruminococcus 2* was positively correlated with ESR, TJC, SJC, DAS28, and anti-CCP, while the downregulated gut microbiota such as *Lachnospira* was negatively associated with RF-IgG (Fig. 4C). Furthermore, *Ruminococcus 2* was identified as the potential gut microbiota biomarker of RA via receiver operating characteristic (ROC) curve analysis for it yielded the highest area under curve (AUC) of the curve [AUC = 0.782, 95% confidence interval (CI) = 0.636–0.929,* P* = 0.001] (Fig. 4D).

### Relationship between altered metabolites and RA

The stool samples of 17 new-onset RA patients and 13 HCs were selected randomly to analyze metabolites by UPLC-MS-based untargeted metabolomic profiling.

**Fig. 5:**
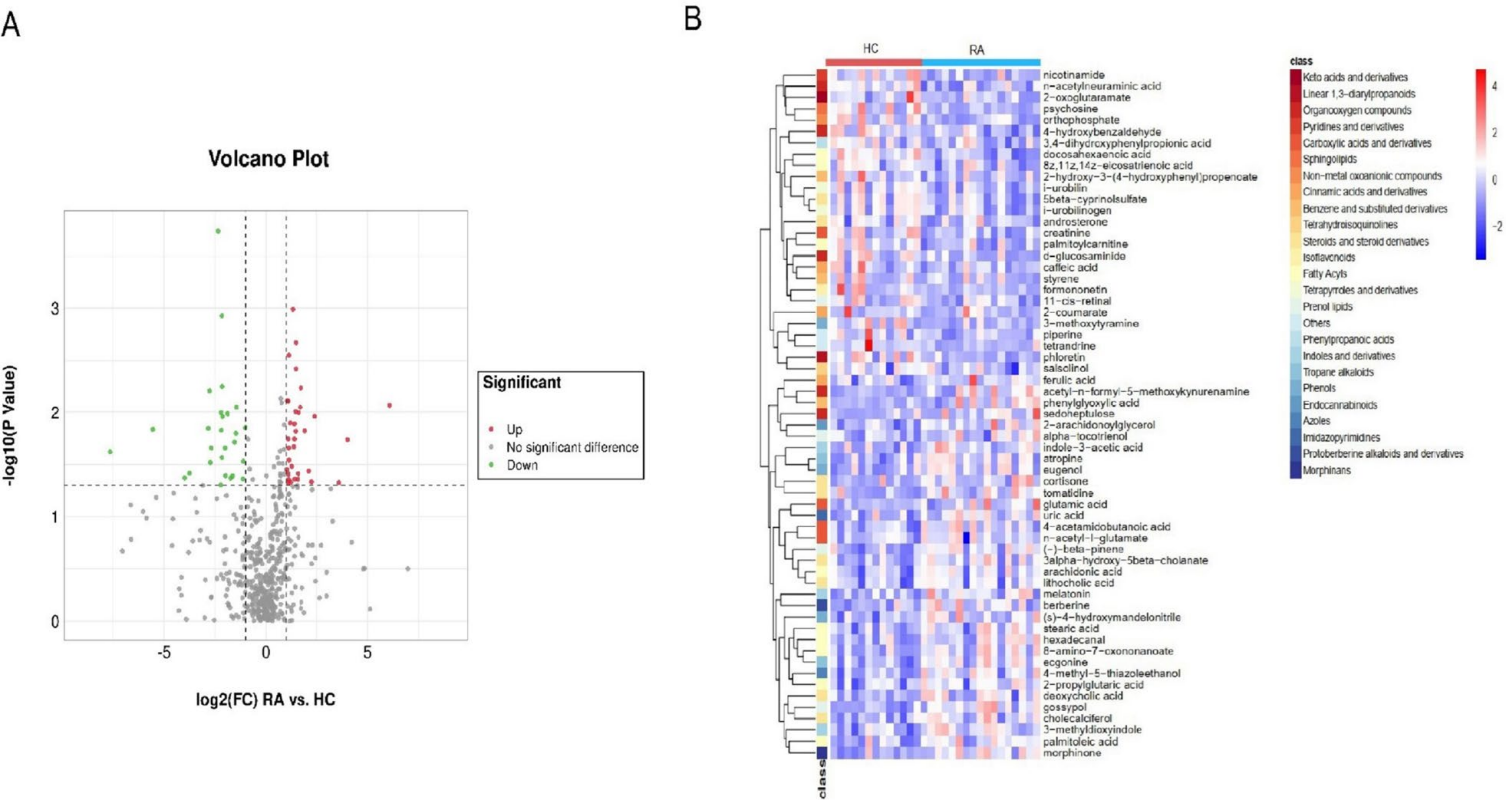
The profiles of gut microbiota-derived metabolites between new-onset patients with RA and HCs. **A** The volcano plot showed the differentially altered metabolites between RA and HCs. The red plot represented the upregulated metabolites, the green plot represented the downregulated metabolites, and the gray plot represented the metabolites with no significant differences. **B** The heatmap of differentially abundant metabolites based on the relative abundance. (RA: rheumatoid arthritis; HCs: healthy controls)

Multivariate statistical analysis was to identify the differential metabolites between the RA and HCs by the projections to latent structures discriminant analysis (PLS-DA) model. It showed that the metabolites between RA and HC were separated by differences in both the positive and negative ion modes. And the permutation test of the PLS-DA model with positive and negative ion modes indicated that the PLS-DA model had a good prediction and explanation ability without overfitting phenomenon (Additional file 1: Fig. S4). It indicated the significant differences in metabolites between new-onset RA and HCs. A total of 61 differential metabolites were recognized including 34 metabolites upregulated and 27 metabolites downregulated compared to HCs according to the conditions of differential metabolites (fold change ≥ 2 or ≤ 0.5, VIP > 1 and *P* < 0.05) (Fig. 5A, B).

**Fig. 6:**
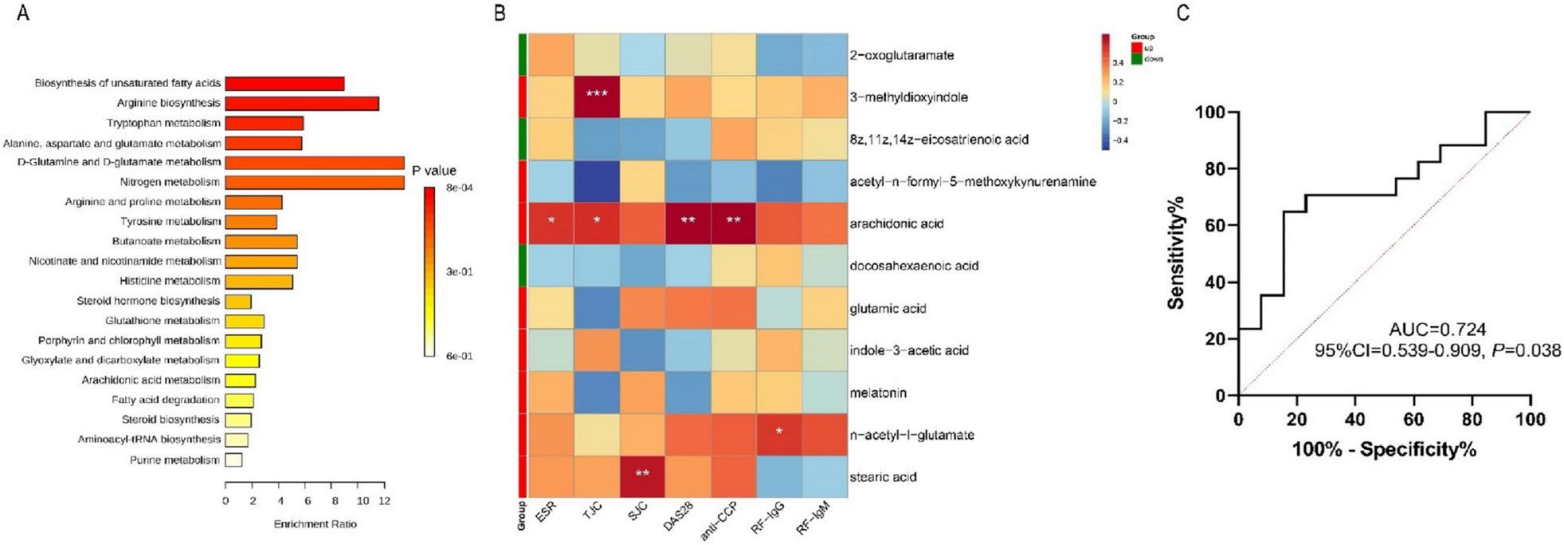
The analysis of gut microbiota-derived metabolites in new-onset patients with RA. **A** The KEGG pathway enrichment analysis of the differentially abundant metabolites. Four pathways including biosynthesis of unsaturated fatty acids (*P* < 0.001), arginine biosynthesis (*P* = 0.012), and tryptophan metabolism (*P* = 0.012), as well as alanine, aspartate, and glutamate metabolism (*P* = 0.004) were the main pathways related to RA in the top twenty pathways. **B** The correlation heatmap of the eleven differentially abundant metabolites involved in the four mainly altered pathway and indicators of RA. **C** The ROC curve of biomarker analysis for arachidonic acid. (RA: rheumatoid arthritis; HCs: healthy controls; KEGG: Kyoto Encyclopedia of Genes and Genomes; ROC: receiver operating characteristic; AUC: area under curve; CI: confidence interval)

Among the top twenty pathways identified by the Kyoto Encyclopedia of Genes and Genomes (KEGG) pathway enrichment analysis of the differentially abundant metabolites, there were only four pathways closely related to RA. It included biosynthesis of unsaturated fatty acids (*P* < 0.001), arginine biosynthesis (*P* = 0.012), and tryptophan metabolism (*P* = 0.012), as well as alanine, aspartate and glutamate metabolism (*P* = 0.004) (Fig. 6A). Within these altered pathways, eleven differentially abundant metabolites were identified including eight abundant in new-onset RA patients while three abundant in HCs (Additional file 1: Table S3).

A correlation heatmap demonstrated the connections between the eleven differentially abundant metabolites and the indicators of RA (Fig. 6B). It showed that the increased arachidonic acid was positively correlated with ESR, TJC, DAS28, and anti-CCP, the increased n-acetyl-l-glutamate was positively correlated with ESR, the increased 3-methyldioxyindole was positively associated with TJC, the increased n-acetyl-l-glutamate was positively associated with RF-IgG, and the increased stearic acid was positively associated with SJC. The above indicated that the differential metabolites in RA were associated with the progression of the disease. And arachidonic acid was identified as the potential metabolite biomarker of RA via ROC curve analysis (AUC = 0.724, 95% CI = 0.595–0.909, *P* = 0.038) (Fig. 6C).

### Altered gut microbiotas and their metabolites in RA were associated with Tfr cells

**Fig. 7:**
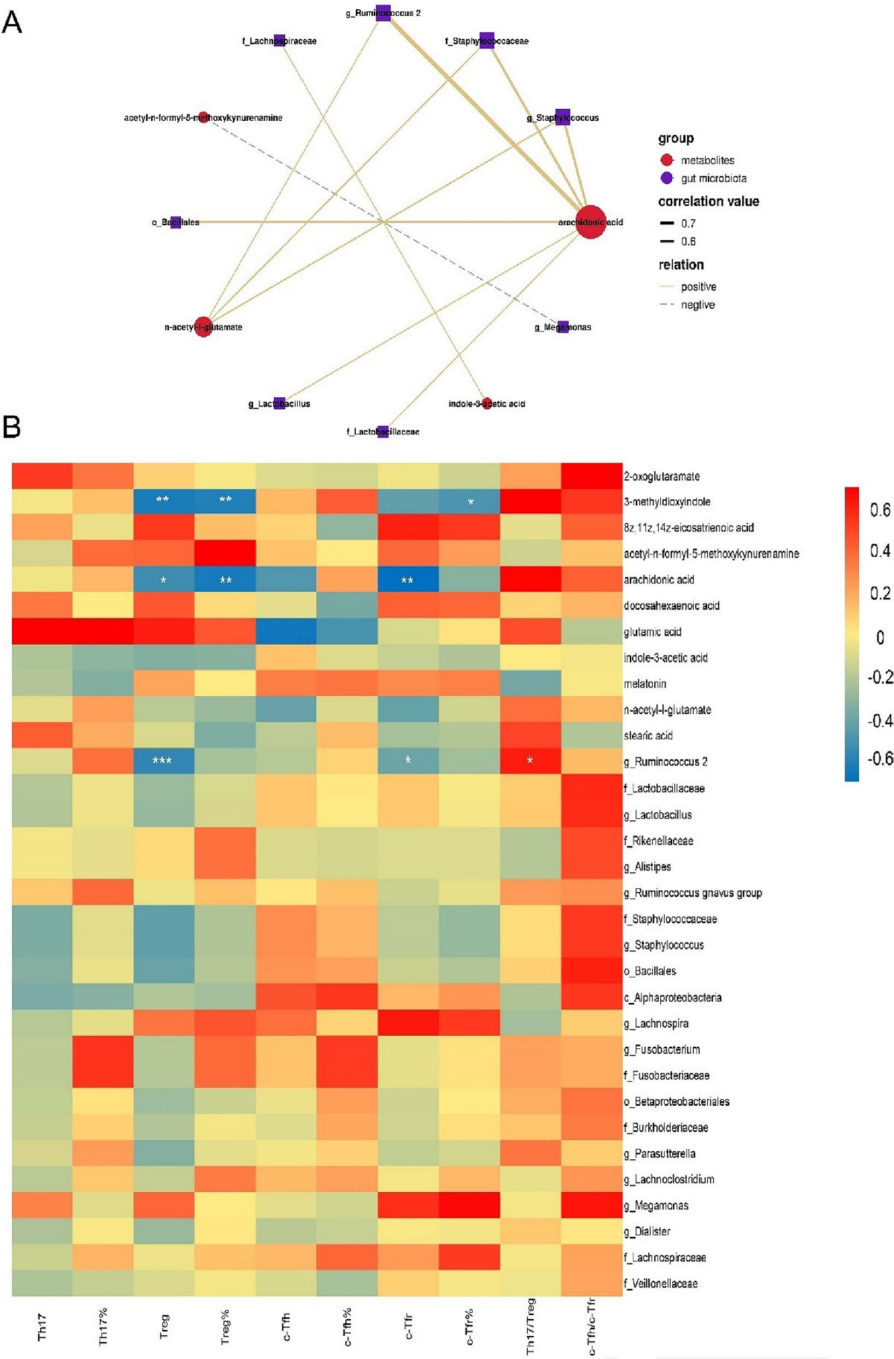
The relationship among the gut microbiota, metabolites and immune cell in RA. **A** The correlation network graph showed the main interactions between gut microbiotas and their metabolites (Spearman’s correlation analysis, r > 0.5, *P* < 0.05). Gut microbiotas were marked in purple and metabolites were marked in red. The Solid connecting lines indicated the positive correlations between gut microbiotas and their metabolites, while dashed connecting lines indicated negative correlations between the two. Thicker lines indicated greater correlation values. It showed that arachidonic acid seemed to be the core metabolite as it was positively associated with six gut microbiotas enriched in RA including *Ruminococcus 2*, *Staphylococcus*, *Staphylococcaceae*, *Bacillales*, *Lactobacillus*, and *Lactobacillaceae*. **B** The correlation heatmap of altered gut microbiotas and their metabolites in RA with the expression of Th17, Treg, c-Tfr, and c-Tfh cells in new-onset RA patients. (RA: rheumatoid arthritis; Th17: helper T 17 cells; Treg: regulatory T cells; Tfr: follicular regulatory T cells; Tfh: follicular helper T cells)

To investigate the interactions between gut microbiotas and their metabolites related to RA, we subsequently assessed the correlations between the 21 differential gut microbiotas and 11 metabolites detected in RA by Spearman’s correlation analysis. The correlation network of the interactions between them showed arachidonic acid was the core metabolite as it was positively associated with six gut microbiotas enriched in RA including *Ruminococcus 2*, *Staphylococcus*, *Staphylococcaceae*, *Bacillales*, *Lactobacillus*, and *Lactobacillaceae* (r > 0.5, *P* < 0.05, Fig. 7A). It indicated that the altered metabolites seemed to be correlated with the gut microbiota dysbiosis in RA.

To further elucidate the relationship of altered gut microbiotas and their metabolites in RA with Treg and Tfr cells, a correlation heatmap was performed (Fig. 7B). In terms of gut microbiota, the increased *Ruminococcus 2* was negatively correlated with the reduced number of Treg and c-Tfr cells but positively associated with the ratio of Th17/Treg. As for the metabolites, the increased arachidonic acid involved in the biosynthesis of unsaturated fatty acid pathway was negatively associated with the reduced level of Treg and c-Tfr cells, and the increased 3-methyldioxyindole involved in the tryptophan metabolism pathway was negatively associated with the reduced Treg and Tfr cells.

## Discussion

Circulating Tfr cells play a role in maintaining immune tolerance, and the reduced Tfr cell may contribute to the breakdown of immune tolerance to participate in the progression of RA. However, the upstream mechanism of regulating Tfr cell-mediated immune tolerance remains unclear. Numerous studies have shown that gut microbiota dysbiosis and altered metabolites are closely related to the development of RA [35, 36], which may be caused by the interactions of gut microbiotas and their metabolites with the immune system. It still lacks systematic studies on the relationship between gut microbiota dysbiosis and altered metabolites with the Tfr cell-mediated immune tolerance in RA. Our study was the first to investigate the association of gut microbiotas and their metabolites with the immune tolerance mediated by Tfr cells in new-onset RA. The results of our study revealed the following characteristics of the new-onset patients with RA: (i) The reduced Treg and Tfr cells in RA were associated with the disease activity and the over-production of autoantibodies. (ii) Gut microbiota dysbiosis (especially at the genus levels) and altered gut microbiota-derived metabolites exhibited in new-onset RA patients were related to the disease. (iii) *Ruminococcus 2* as well as arachidonic acid might be the potential biomarkers of RA. (iv) Gut microbiota interacted with their metabolites, and gut microbiota dysbiosis as well as the altered metabolites in RA were associated with the breakdown of immune tolerance mediated by reduced Tfr cells. The association between gut microbiotas and their metabolites with immune tolerance mediated by Tfr cells we pointed out may participate in RA, which provided a theoretical basis for further exploring the effect of specific gut microbiota and its metabolites on Tfr cells.

Early work suggested that the activated CD4 + CD25 + CD69 − Treg cells were able to gain the expression of CXCR5 and migrate to the B-cell follicle to suppress B-cell responses [41, 42], which revealed the relationship between Treg cells and another new type of cells in lymphoid follicular. And then, three independent groups defined them as Tfr cells (CXCR5 + PD-1 + BCL6 + FoxP3 + cells), which originated de novo from thymic-derived FoxP3 + Treg precursors requiring multiple stimulations [13–15]. Increasing data highlighted the significance of Treg and Tfr cells in maintaining immune tolerance, especially Tfr cells, which exert an essential effect on regulating antibody production as the subsets of Treg cells via suppressing Tfh cells and B cells in GCs [16]. Considering the difficulties of getting organ tissues from humans for clinical investigations, the circulating Tfr and Tfh cells are always discussed in regulating immune tolerance, which may be derived from GCs and have similar phenotypes and function to GC-Tfh and GC-Tfh cells [43]. By analyzing the expression of circulating Th17, Treg, Tfr, and Tfh cells, our study found that in the new-onset patients with RA, the ratio of Tfh/Tfr was aberrant, and Treg and c-Tfr cells were reduced and associated with the disease activity and the abnormal autoantibodies of RA negatively, but the expressions of Th17 and c-Tfh cells were not increased significantly. The results were consistent with the previous studies [9, 18–20] and supported the previous spot that the immune tolerance breakdown mediated by reduced Treg and Tfr cells participated in the progression of RA rather than the over-immune response. Interestingly, our study found that both Treg and Tfr cells were decreased in RA and were related to disease activity and antibody production of RA, but Treg cells were mainly related to disease activity while Tfr cells were mainly related to antibody production specifically. The above might suggest that the function of Treg cells and Tfr cells in regulating antibody production and maintaining immune tolerance was different despite that Tfr cells were derived from Treg cells. The role of Tfr cells in the early stage of RA, even Pre-RA, needs further exploration.

The interactions between gut microbiotas and their metabolites with the host are important in health and disease, especially the role of gut microbiotas and their metabolites in RA is the focus of our research. Our study revealed that gut microbiota was dysbiosis in new-onset RA patients. Especially the increased *Ruminococcus 2*, one of the predominant gut microbiotas in RA, exhibited a positive correlation with disease activity and autoantibody production. And *Ruminococcus 2* might be the potential biomarker of RA. Notably, our study found that *Lachnospira* was recognized as one of the 21 differential gut microbiotas from phylum to genus levels between RA and HCs, and it was downregulated in new-onset RA patients and negatively associated with RF-IgG of RA. *Lachnospira* is mainly present in the gut of most healthy individuals and may be a potential probiotic involved in the metabolism of a variety of carbohydrates. A two-sample Mendelian randomization study of the causal effects between gut microbiome and systemic lupus erythematosus (SLE) showed that *Lachnospira* was negatively correlated with the risk of SLE [44]. Although our study found the reduced *Lachnospira* in RA was only related to RF-IgG negatively, it still suggested that the reduced *Lachnospira* might promote RA, and the supplementation of it may be a potential treatment for RA. Of course, it still needs further exploration. Additionally, our results also showed that the gut microbiota-derived metabolites involved in the biosynthesis of unsaturated fatty acids, arginine biosynthesis, and tryptophan metabolism as well as alanine, aspartate, and glutamate metabolism were altered in RA, which was consistent with the prior studies [35, 36, 45]. We found that the altered metabolites involved in the biosynthesis of unsaturated fatty acids and tryptophan metabolism pathways were associated with the progression of RA. Especially, the increased arachidonic acid in RA showed positive correlations with disease activity and autoantibody production. And it exhibited good discrimination in distinguishing RA and HCs as the potential biomarker value of RA. In brief, the above supported that gut microbiota dysbiosis and altered metabolites in RA were associated with the development of RA.

Gut microbiotas and their metabolites are the vital bridge of the gut-joint axis to contribute to the pathogenesis of RA [26, 34, 46], but the mechanism remains unclear. Exploring the mechanism of RA triggered by gut microbiotas and their metabolites through the gut-joint axis has great significance. The trafficking of activated pro-inflammatory immune cells from the gut to joints has been thought to be one of the main mechanisms of driving the RA onset through the gut-joint axis [37, 38]. T cells in the synovium of patients with RA have been found to express the gut-homing receptor αEβ7 integrin supporting the viewpoint that the trafficking of mucosa-derived immune cells (such as mucosal-associated invariant T cells, Th17 cells, γδT cells, Tfh cells, and so on) from gut to joints [37, 38]. Gut-residing segmented filamentous bacteria (SFB) was found to promote autoimmune arthritis in K/BxN mouse models via the migration of Tfh cells and Th17 cells suggesting the interactions of gut microbiota and effector T cells contribute to the development of RA [47, 48]. Meanwhile, it is worth noting that the gut microbiotas and their metabolites may also contribute to the pathogenesis of RA by regulating Treg and Tfr cell-mediated immune tolerance through the gut-joint axis.

The interactions between gut microbiotas and Treg cells have attracted extensive attention, which is important in establishing intestinal immune tolerance. On the one hand, gut microbiotas could regulate the function and expression of Treg cells by influencing the Treg cell-modulatory activity (such as transforming growth factor-β) directly or controlling signals coming from epithelial cells, dendritic cells, or other Treg cell-regulating cells indirectly [49, 50]. On the other hand, Treg cells exert the effect in establishing intestinal immune homeostasis by inducing the tolerance to symbiotic flora and the host defense against intestinal pathogens, and gut microbial-specific Treg cells have been confirmed to be the essential cells to induce intestinal immune tolerance [51–53]. The effects of gut microbiotas on Treg cells are also researched in RA. The association of gut microbiota dysbiosis and reduced Treg cells in RA patients has been observed [54, 55]. And the animal experiments also confirmed that gut microbiotas and their metabolites affected Treg cells [56–58]. The reduced *Bacteroides fragilis* in collagen-induced arthritis (CIA) mice inhibited the differentiation of CD4 + T cells into Treg cells, while the colonization of *Bacteroides fragilis* in germ-free mice promoted the proliferation of Treg cells and the production of anti-inflammatory cytokines [56, 57]. *Lactobacillus casei* CCFM1074 strain also upregulated the number of Treg cells in the CIA mouse model [58]. In addition, as one of the most abundant gut microbiota-derived metabolites, SCFAs could also regulate the Treg cell-mediated immune tolerance by upregulating the expression of Foxp3 in Treg cells and enhancing the ability of dendritic cells to induce differentiation of Treg cells [59, 60]. Butyrate as the component of SCFA, it has been found that patients with RA lacked the butyrate-producing species and the supplementation of dietary butyrate could exert anti-inflammatory effects to ameliorate RA by promoting Treg cells while suppressing effector T cells and osteoclasts [39]. The above evidence suggests that gut microbiota dysbiosis and altered metabolites may contribute to RA by influencing immune tolerance mediated via Treg cells, which seems to be one of the mechanisms of the gut-joint axis. Tfr cells are largely derived from Treg cells; therefore, Tfr cells are also the potential targets for gut microbiotas and their metabolites to regulate immune tolerance in patients with RA. However, few studies have revealed the relationship of gut microbiotas and their metabolites with Tfr cells directly. Although there had been some studies finding that arthritis induced by SFB was associated with the reduced expression of cytotoxic T-lymphocyte-associated protein 4 (CTLA-4) on the surface of Tfr cells [61] and microbiota-derived butyrate could suppress the development of autoimmune arthritis by enhancing the histone acetylation of Tfr cell to promote their differentiation [40], the effects of gut microbiotas and their metabolites on Tfr cells still value the in-depth study in the future, especially to explore the role of other microbiotas and metabolites on regulating Tfr cells to participate in RA. Our study was the first to investigate the association of gut microbiotas and their metabolites with immune tolerance mediated by Tfr cells and found that gut microbiota dysbiosis and altered metabolites were related to the reduced Treg and Tfr cells. Specifically, the increased *Ruminococcus 2*, the increased arachidonic acid involved in the biosynthesis of unsaturated fatty acid pathway and the increased 3-methyldioxyindole involved in the tryptophan metabolism pathway exhibited negative correlations with the reduced Treg and Tfr cells. It meant that RA patients with altered gut microbiotas and their metabolites were more likely to exhibit impaired immune tolerance mediated by reduced Tfr cells. Therefore, we suspected that the association of altered gut microbiotas and their metabolites in RA with the breakdown of immune tolerance mediated by the reduced Tfr cells was involved in the development of RA.

*Ruminococcus* is a kind of common commensal gut microbiota present in healthy individuals with low abundance, and the increase of it would directly lead to the disruption of intestinal barrier function [62], which may be involved in the pathogenesis of autoimmune diseases. Some studies have shown that *Ruminococcus* was elevated in SLE [63] and spondyloarthropathies [64]. *Ruminococcus* was also found to be positively correlated with RF-IgA and anti-CCP antibodies and the disease activity of RA [65, 66]. And the deletion of T-cell death-associated gene 8 (TDAG8) was found to significantly reduce local mucosal inflammation and relieve the disease severity of RA by decreasing the abundance of proinflammation-related *Ruminococcus* [67], which both suggested the important role of *Ruminococcus* in RA. Here, we focused on *Ruminococcus 2*, which was one of the abundant gut microbiotas detected in RA and was associated with the progression of RA. The role of *Ruminococcus 2* in RA has not been well-studied. Only one study has found that *Ruminococcus 2* was more abundant in RA patients with lower Treg cells indicating that *Ruminococcus 2* was associated with Treg cells in RA [54]. And even fewer studies about *Ruminococcus 2* and Tfr cells. Based on our results, we hypothesized that individuals with specific compositions of gut microbiota such as increased *Ruminococcus 2* might be more susceptible to RA by reducing Tfr cells to destroy immune tolerance. Arachidonic acid, a polyunsaturated fatty acid, is an important inflammatory mediator in exerting regulatory effects as the direct precursor of various bioactive lipid mediators [68] and active substances such as prostaglandin E2, prostaglandin I2, and thromboxane A2 [69]. A recent study of the serum metabolites in Pre-RA showed that arachidonic acid was enriched in the Pre-RA group [45], and there also had been some studies about the role of arachidonic acid in RA [70, 71]. It showed that arachidonic acid could regulate calcium signaling in the T cells of patients with RA to promote synovial inflammation [70]. And further study showed that *Ershiwuwei Lvxue Pill* (ELP), a prescription of Tibetan medicine, could alleviate cartilage and bone injury by regulating host metabolites such as arachidonic acid [71]. The results of our study supported that arachidonic acid was the core metabolite of gut microbiota and the increase of it was associated with reduced Treg and Tfr cells. We suspected that the increased arachidonic acid in RA patients may be mainly caused by the gut microbiota dysbiosis, and it promoted the conversion of the immune balance towards autoimmunity to contribute to RA, which is related to the breakdown of immune tolerance mediated by reduced Tfr cells. In addition, our study showed that *Ruminococcus 2* and arachidonic acid were positively related with each other and they were both associated with the symptoms of arthritis as the potential biomarker of RA. The increased *Ruminococcus 2* might aggravate arthritis and pain by promoting the production of arachidonic acid to further generate the active substances including prostaglandin.

Additionally, tryptophan metabolism could exert effects in regulating the immune system, and it has also been confirmed that the abnormal tryptophan metabolism was related to autoimmune disease [72–74]. There are three main metabolic pathways for tryptophan, and nearly 90% of tryptophan is metabolized by indoleamine-2,3-dioxygenase (IDO) to produce intermediate metabolite kynurenine [75]. And CD4 + CD25 − T cells can be transformed into Foxp3 + CD4 + CD25 + Treg cells through IDO-mediated tryptophan metabolism suggesting the role of tryptophan metabolism in regulating immune tolerance [76]. Gut microbiota participates in tryptophan metabolism directly or indirectly, and gut microbiota-derived tryptophan metabolites were found to be associated with autoimmune arthritis [72, 73]. IDO is highly expressed in intestinal epithelial cells, which may be affected by gut microbiota to further influence tryptophan metabolism and the transformation of Treg cells [77]. The interactions between gut microbiota and tryptophan metabolism may regulate Treg cell-mediated immune tolerance, but the effect on Tfr cells remains unclear. Considering that Tfr cells was differentiated from Treg cells, the tryptophan metabolism affected by gut microbiota might also regulate Tfr cells, which needed further exploration. Our study suggested that there was an altered distribution of fecal tryptophan metabolites of new-onset RA and they were correlated with the arthritis symptoms and the reduced Treg and Tfr cells. It provided support for exploring the effect of tryptophan metabolism on immune cells, especially Tfr cells, in RA. However, the exact effect of tryptophan metabolism on Tfr cells needs further exploration.

Our result elucidated that gut microbiotas and their metabolites were potential disease markers for RA, and there was a correlation between them with Treg and Tfr cells. The influence of gut microbiota, especially their metabolites, on immune cells is still a frontier of immunomicroecology. The effects of gut microbiota-derived metabolites including SCFAs, bile acids, and tryptophan and its derivatives on Treg cells have been demonstrated, and previous studies have also found that SCFA affected Tfr cells. Our study highlighted the association of the biosynthetic unsaturated fatty acid pathway and the tryptophan metabolic pathway with Tfr cells, which provided valuable insights for exploring the effect of other metabolites on the immune tolerance of RA in the future, especially tryptophan metabolism. Understanding these relationships may help to develop the potential therapeutic strategies targeted at modulating gut microbiotas and their metabolites to restore immune tolerance and improve RA management. However, there were some limits in our study. Firstly, the sample sizes were small and limited to a single center, which may affect the generalizability of the results. Larger and multi-center studies are needed to further verify the results, and it was also necessary to enroll in the different stages of RA (including pre-clinical RA, RA transition, early RA, and established RA) to further explore the characteristics of gut microbiotas and their metabolites during the progression of RA. Secondly, as an observational study, our study only demonstrated the relationship of gut microbiotas and their metabolites with Tfr cells in RA instead of the causality, it is necessary to conduct the vitro experiments to elucidate the causal relationship between them and identify whether the gut microbiotas and their metabolites contribute to the pathogenesis of RA by influencing Tfr cells as one of the mechanisms involved in the gut-joint axis. Finally, further studies are necessary to confirm the specific biological significance underlying these differences in the identified metabolites in RA, for example targeting the metabolism of tryptophan and unsaturated fatty acids may have significance in exploring the pathogenesis of RA.

## Conclusion

Our study revealed that gut microbiota dysbiosis and altered metabolites were associated with the breakdown of immune tolerance mediated by reduced Tfr cells in RA. Based on it, we hypothesized that the altered gut microbiotas and their metabolites might result in the breakdown of immune tolerance by affecting Tfr cells. This finding highlighted the importance of gut microbiotas and their metabolites in the gut-joint axis. It also provides a foundation for further investigations into the potential role of gut microbiotas and their metabolites on Tfr cell-mediated immune tolerance in RA from the perspective of microecology-metabolism-immune. This could help us to better understand the pathogenesis of RA and develop new treatments for the disease.

### Supplementary Information

**Additional file 1: Methods. Table S1.** The comparison in the expression of Th17, Treg, Tfr and Tfh cells in the peripheral blood between the patients with RA and HCs. **Table S2.** The comparison of the gut microbiotas with significant differences at the phylum and genus level between the new-onset RA patients and HCs. **Table S3.** The differentially abundant metabolites involved in the four mainly altered pathways. **Fig. S1.** The representative flow cytometry analysis of circulating Th17, Treg, Tfr and Tfh cells. (A) The circulating Th17 cells were identified as CD4+IL-17+T cells. (B) The circulating Treg cells were identified as CD4+CD25+FoxP3+T cells. (C) The circulating Tfh cells were identified as CD3+CD4+CXCR5+CD45RA-PD-1+ T cells. (D) The circulating Tfr cells were identified as CD3+CD4+ CXCR5+ CD45RA- CD25+ FoxP3+cells (Th17: helper T 17 cells; Treg: regulatory T cells; Tfr: follicular regulatory T cells; Tfh: follicular helper T cells). **Fig. S2.** The correlation between the number of Treg cells and Tfr cells in the new-onset RA patients. The two cells were related positively. (Treg: regulatory T cells; Tfr: follicular regulatory T cells; CI: confidence interval). **Fig. S3.** The α-diversity analysis of new-onset RA patients and HCs. It was assessed by (A) Chao1, (B) Observed species, (C) Shannon and (D)Simpson indicators and showed that the species richness and evenness of gut microbiota in the new-onset RA patients were similar with that in HCs. (RA: rheumatoid arthritis; HCs: healthy controls). **Fig. S4.** Multivariate statistical analysis of fecal metabolite profiles between the RA and HCs. (A) The PLS-DA model showed that the fecal metabolites between the RA and HC were separated by differences in the positive ion mode. (B) The validation model of the PLS-DA model in the positive ion mode indicating that the PLS-DA model had a good ability of prediction and explanation without overfitting phenomenon (Intercept of R2 = 0.8908, Intercept of Q2 = -0.3216). (C) The PLS-DA model showed that the fecal metabolites between the RA and HC were separated by differences in the negative ion mode. (D) The validation model of the PLS-DA model in the negative ion mode indicating that the PLS-DA model had a good ability of prediction and explanation without overfitting phenomenon (Intercept of R2 = 0.909, Intercept of Q2 = -0.2835). (RA: rheumatoid arthritis; HCs: healthy controls; LDA: linear discriminant analysis; PLS-DA: projections to latent structures discriminant analysis).

## Data Availability

The datasets of gut microbiota generated for this study can be found in the SRA of NCBI: https://www.ncbi.nlm.nih.gov/sra/ PRJNA955157 (Temporary Submission ID: SUB13057507). The other data can be provided by the corresponding author and requests for the data should be submitted to snwch@sina.com.

## Abbreviations

RA: Rheumatoid arthritis
anti-CCP: Anti-cyclic peptide containing citrulline
Pre-RA: Pre-clinical RA
FoxP3: Forkhead Box 3
Treg cell: Regulatory T cell
Tfr cell: Follicular regulatory T cell
Th 17 cell: Helper T 17 cell
GC: Germinal centers
Tfh cell: Follicular helper T cell
UPLC-MS: Ultra-performance liquid chromatography-tandem mass spectrometry
DMARDs: Disease-modifying antirheumatic drugs
HC: Healthy control
TJC: Tenderness joint count
SJC: Swollen joint count
DAS28: Disease activity score 28
ESR: Erythrocyte sedimentation rate
RF: Rheumatoid factor
SD: Standard deviation
PCoA: Principal coordinate analysis
ANOSIM: Analysis of similarities
LDA: Linear discriminant analysis
ROC: Receiver operating characteristic
AUC: Area under curve
CI: Confidence interval
PLS-DA: Projections to latent structures discriminant analysis
KEGG: Kyoto Encyclopedia of Genes and Genomes
CXCR5: C-X-C chemokine receptor type 5
SFB: Segmented filamentous bacteria
SCFAs: Short-chain fatty acids
CTLA-4: Cytotoxic T-lymphocyte-associated protein 4
CIA: Collagen-induced arthritis
IDO: Indoleamine-2,3-dioxygenase
