# Supplementary material for: Impaired immune tolerance mediated by reduced Tfr cells in rheumatoid arthritis linked to gut microbiota dysbiosis and altered metabolites
Source: Arthritis Res Ther. 2024 Jan 13;26:21. doi: 10.1186/s13075-023-03260-y (PMC10787489; doi:10.1186/s13075-023-03260-y)
Supplement: Supplementary file 1 — Additional file 1: Methods. Table S1. The comparison in the expression of Th17, Treg, Tfr and Tfh cells in the peripheral blood between the patients with RA and HCs. Table S2. The comparison of the gut microbiotas with significant differences at the phylum and genus level between the new-onset RA patients and HCs. Table S3. The differentially abundant metabolites involved in the four mainly altered pathways. Fig. S1. The representative flow cytometry analysis of circulating Th17, Treg, Tfr and Tfh cells. (A) The circulating Th17 cells were identified as CD4+IL-17+T cells. (B) The circulating Treg cells were identified as CD4+CD25+FoxP3+T cells. (C) The circulating Tfh cells were identified as CD3+CD4+CXCR5+CD45RA-PD-1+ T cells. (D) The circulating Tfr cells were identified as CD3+CD4+ CXCR5+ CD45RA- CD25+ FoxP3+cells (Th17: helper T 17 cells; Treg: regulatory T cells; Tfr: follicular regulatory T cells; Tfh: follicular helper T cells). Fig. S2. The correlation between the number of Treg cells and Tfr cells in the new-onset RA patients. The two cells were related positively. (Treg: regulatory T cells; Tfr: follicular regulatory T cells; CI: confidence interval). Fig. S3. The α-diversity analysis of new-onset RA patients and HCs. It was assessed by (A) Chao1, (B) Observed species, (C) Shannon and (D)Simpson indicators and showed that the species richness and evenness of gut microbiota in the new-onset RA patients were similar with that in HCs. (RA: rheumatoid arthritis; HCs: healthy controls). Fig. S4. Multivariate statistical analysis of fecal metabolite profiles between the RA and HCs. (A) The PLS-DA model showed that the fecal metabolites between the RA and HC were separated by differences in the positive ion mode. (B) The validation model of the PLS-DA model in the positive ion mode indicating that the PLS-DA model had a good ability of prediction and explanation without overfitting phenomenon (Intercept of R2 = 0.8908, Intercept of Q2 = -0.3216). (C) The PLS-DA m [file 13075_2023_3260_MOESM1_ESM.docx]

**Supplementary Material**

**Methods**

***1.1. The detection of Th17, Treg, Tfr and Tfh cells***

Firstly, the absolute number of CD4+ T cells was measured. 20μl of anti-CD3 /CD8/CD45/CD4 reagent was added to the bottom of the BD TrucountTM test tube containing magnetic beads. 50μl of EDTA anticoagulant venous blood was added and mixed gently by shaking. After incubation at 20-25℃ for 15min, 450μl of diluted 1X hemolysin was added to the mixture, and the test tube was kept away from the light for 15-20min at 20-25℃.

And next was to detect the expression of Th17 and Treg cells. 200μl of blood sample, 200μl of RPMI-1640 and 1μl of leukocyte activation kit regent were mixed in a test tube, which was them incubated at 37°C under 5% CO2 for 5 hours to detect the expression of Th17 cells. Then, anti-CD4-FITC was added into the tube to stain at room temperature in the dark for 30 minutes after the lysis of red blood cells. And then Fixation/Permeabilization was added into the test tube to place at 4°C in the dark for 30 minutes. Finally, anti-IL-17-PE was then added for intracellular staining in the dark for 30 minutes. As for the detection of Treg cells, anti-CD4-FITC and anti-CD25-APC were added into 100μl of blood sample after the lysis of red blood cells, and then it was incubated at room temperature in the dark for 30 minutes. Next, the test tube was incubated at 4°C for 30 min after adding fixation/permeabilization buffer, and anti-FoxP3-PE was then added for intracellular staining for 30 minutes in the dark in final.

Then, two tubes (tube A and tube B) were prepared to detect the expression of Tfr and Tfh cells respectively. Each tube was filled with 200μl of EDTA anticoagulant venous blood. For tube A, 2.5μl CXCR5, 2.5μl CD3, 2.5μl CD4, 10μl CD45RA, and 2.5μl CD25 were added to stain the blood at room temperature for 30 minutes away from light to detect the expression of c-Tfr cells. Following the lysis of red blood cells by 2ml diluted 1X hemolysin, 1ml of Fixation/Permeabilization was added to tube A. After 30min at room temperature, 2.5μl of FoxP3 was added for intracellular staining for 30min at room temperature and away from light, following one wash with 2ml of diluted 1X Buffer. Then 2ml of diluted 1X Buffer was added and washed again. Finally, 400μl diluted 1X Buffer was added for the test on the flow cytometry. For tube B, 2.5μl CXCR5, 2.5μl CD3, 2.5μl CD4, 10μl CD45RA, and 10μl PD-1 were added to stain the blood at room temperature for 30 minutes away from light to detect the expression of c-Tfh cells. Then 2 ml normal saline was added to wash after the lysis of red blood cells by 2ml diluted 1X hemolysin. And finally, 300μl normal saline was added for the test on the flow cytometry.

The relative percentages of Th17, Treg, c-Tfr and c-Tfh cells in CD4+T cells were analyzed by Cell Quest software, and the number of these cells was calculated by multiplying the relative percentages by the number of CD4+ T cells, which was determined using BD Trucount™ tubes containing magnetic beads. All the tubes were washed with phosphate buffer saline (PBS) and tested on the flow cytometer (FACSCanto^TM^ II; Becton Dickinson, San Jose, California, USA) within 2h. All antibody reagents were from BD Biosciences.

***1.2. The analysis of gut microbiota***

The total DNA of the pretreated 200mg fecal samples was extracted strictly following the instructions of E.Z.N.A. ^®^Stool DNA Kit (D4015, Omega, Inc., USA). The sequencing region of the 16S rRNA gene was the highly variable region(V3-V4). And it was amplified by polymerase chain reaction (PCR). The PCR primer was designed against the conserved region to target the variable region of the 16S rDNA gene with the forward primer (341F) 5’-CCTACGGGNGGCWGCAG-3’ and reverse prime r(805R) 5’-GACTACHVGGGTATCTAATCC-3’. 2% agarose gel was used to detect the amplification products, and then PCR products were purified and quantified. The hybrid library was constructed and denatured into single chain by sodium hydroxide. The pooled library was loaded on the Illumina platform using a paired-end sequencing protocol (2 x 250 bp). Paired-end reads were merged via FLASH (v1.2.8). High-quality clean data were obtained by the FQTrim (v0.94). And feature data were obtained using DADA2, which were compared with the SILVA and NT-16S databases for microbial identification and annotation.

The *α* diversity and *β* diversity were analyzed by QIIME2. Unweighted UniFrac distance served as the foundation for the principal coordinate analysis (PCoA), and the permutation test was used to determine the *P*-value of the analysis of similarities (ANOSIM). The Wilcoxon test and linear discriminant analysis (LDA) effect size (LEfSe) analysis were conducted to determine differential taxa. Graphs were produced using R4.1.0.

***1.3. The analysis of metabolites***

The 50mg fecal samples were ground with liquid nitrogen and then 120ul of 50% methanol was added to the sample. Next, the sample was vortexed thoroughly and incubated at room temperature for 10 min to extract the metabolites. the extract solution was put at -20°C temperature overnight to precipitate the protein in the sample. The metabolite extract solution of the supernatant was transferred into 96-well plate after the centrifugation at 4000g for 20 minutes. 10ul diluent was taken from each mixed sample to form QC samples. All metabolic samples were stored in -80°C until the subsequent high-resolution mass spectrometry analysis, which was performed by the UPLC‐MS system (TripleTOF5600plus, SCIEX, UK) following machine instructions.

Each sample was both collected in positive ion mode and in negative ion mode, and the Ionspray voltage floating were set at 5000 V and ‐ 4500V respectively. The MS data were acquired in Information Dependent Acquisition (IDA) mode. XCMS software was used to preprocess the collected MS data. The LC-MS raw data files were converted to mzXML format and then processed by CAMERA and metaX toolbox. By integrating retention time (RT) and m/z data, each ion was identified. Each peak's intensities were noted, and a three-dimensional matrix was created with randomly assigned peak indices (RTm/z pairs), sample names (observations), and information about ion intensity (variables). The online Human Metabolome Database (HMDB) and Kyoto Encyclopedia of Genes and Genomes (KEGG) were used to annotate the metabolites by matching the precise molecular mass data (m/z) of samples with those from the database. An in‐house fragment spectrum library of metabolites was also used to validate the identified metabolites. Data on metabolites were standardized and quantified.

The extraction and analysis of fecal metabolites were listed in supplementary materials. Student’s t-test, fold change (FC) values and multivariate analysis including projections to latent structures discriminant analysis (PLS-DA) were used to screen the differential metabolites. The variable importance for the projection (VIP) value was calculated by PLS-DA. The metabolites with FC ≥ 2 or ≤ 0.5, VIP > 1 and P < 0.05 were screened as the differential metabolites.

| **Table S1.** The comparison in the expression of Th17, Treg, Tfr and Tfh cells in the peripheral blood between the patients with RA and HCs. | | | |
| --- | --- | --- | --- |
|  | **New-onset RA patients**  **(n=32)** | **HC**  **(n=17)** | ***P* value** |
| **The number of cells(cells/µl)** | | | |
| CD4+ T cells | 652.110(470.295,907.850) | 772.450(644.190,960.625) | 0.153 |
| Th17 cells | 8.110(4.308,10.813) | 9.670(5.760,12.030) | 0.248 |
| Treg cells | 20.605(15.633,33.415) | 35.700(23.360,59.855) | 0.004** |
| Th17/Treg | 0.305(0.226,0.540) | 0.230(0.130,0.415) | 0.106 |
| Tfh cells | 62.214(34.320,95.087) | 28.662(8.302,82.789) | 0.074 |
| Tfr cells | 7.690(1.700,15.202) | 14.519(8.979,28.602) | 0.008** |
| Tfh/Tfr | 7.822(3.546,26.824) | 1.916(0.684,4.483) | <0.001*** |
| **The percent of cells (%)** | | | |
| Th17 cells | 1.125(0.740,1.545) | 1.070(0.495,1.255) | 0.323 |
| Treg cells | 3.275(2.555,4.593) | 3.640(2.865,5.835) | 0.189 |
| Tfh cells | 8.955(5.580,13.550) | 2.750(0.923,12.965) | 0.044* |
| Tfr cells | 1.332(0.282,2.224) | 2.097(1.143,3.130) | 0.023* |
| Results were expressed as the median (Q1, Q3), and were analyzed by Mann–Whitney *U* test.  **P* < 0.05, ***P*< 0.01, ****P*< 0.001  Abbreviations: RA: rheumatoid arthritis; HC: healthy control; Th17: helper T 17 cells; Treg: regulatory T cells; Tfr: follicular regulatory T cells; Tfh: follicular helper T cells. | | | |

| **Table S2.** The comparison of the gut microbiotas with significant differences at the phylum and genus level between the new-onset RA patients and HCs. | | | |
| --- | --- | --- | --- |
|  | **New-onset RA patients**  **(n=32)** | **HC**  **(n=17)** | ***P* value** |
| **Phylum** | | | |
| *Nitrospirae* | 0.017(0.013,0.025) | 0.009(0.000,0.017) | 0.007** |
| *Spirochaetes* | 0.000(0.000,0.061) | 0.000(0.000,0.000) | 0.007** |
| *Bacteroidota* | 0.078(0.004,0.114) | 0.000(0.000,0.061) | 0.017* |
| **Genus** | | | |
| *Ruminococcus_2* | 1.342(0.294,6.645) | 0.101(0.049,1.600) | 0.001** |
| *Lactobacillus* | 1.520(1.037,1.973) | 1.047(0.788,1.213) | 0.004** |
| *Alistipes* | 0.814(0.141,2.003) | 0.118(0.014,0.554) | 0.044* |
| *Dialister* | 0.123(0.037,0.830) | 2.340(0.165,3.703) | 0.003** |
| *Ruminococcus gnavus group* | 0.057(0.023,0.180) | 0.103(0.073,0.554) | 0.039* |
| *Megamonas* | 0.002(0.000,0.031) | 0.059(0.021,0.114) | 0.003** |
| *Lachnoclostridium* | 0.309(0.132,0.620) | 0.910(0.299,1.861) | 0.007** |
| Results were expressed as the median (Q1, Q3), and were analyzed by Mann–Whitney *U* test.  **P* < 0.05, ***P*< 0.01, ****P*< 0.001  Abbreviations: RA: rheumatoid arthritis; HC: healthy control. | | | |

| **Table S3.** The differentially abundant metabolites involved in the four mainly altered pathways. | | | |
| --- | --- | --- | --- |
|  | Ratio  (RA *vs.* HC) | *P* value | Regulate |
| **Biosynthesis of unsaturated fatty acids pathway** | | | |
| arachidonic acid | 2.259 | 0.013* | up |
| stearic acid | 2.0211 | 0.035* | up |
| docosahexaenoic acid | 0.196 | <0.001*** | down |
| 8z,11z,14z-eicosatrienoic acid | 0.497 | 0.014* | down |
| **Arginine biosynthesis pathway** | | | |
| n-acetyl-l-glutamate | 2.175 | 0.023* | up |
| glutamic acid | 4.367 | 0.036* | up |
| **Tryptophan metabolism pathway** | | | |
| 3-methyldioxyindole | 3.043 | 0.010* | up |
| acetyl-n-formyl-5-methoxykynurenamine | 67.717 | 0.009** | up |
| indole-3-acetic acid | 2.152 | 0.022* | up |
| melatonin | 2.131 | 0.039* | up |
| **Aspartate and glutamate metabolism pathway** | | | |
| 2-oxoglutaramate | 0.252 | 0.040* | down |
| Results were analyzed by Student *t* test. **P* < 0.05, ***P*< 0.01, ****P*< 0.001  Abbreviations: HC: healthy control; RA: rheumatoid arthritis. | | | |


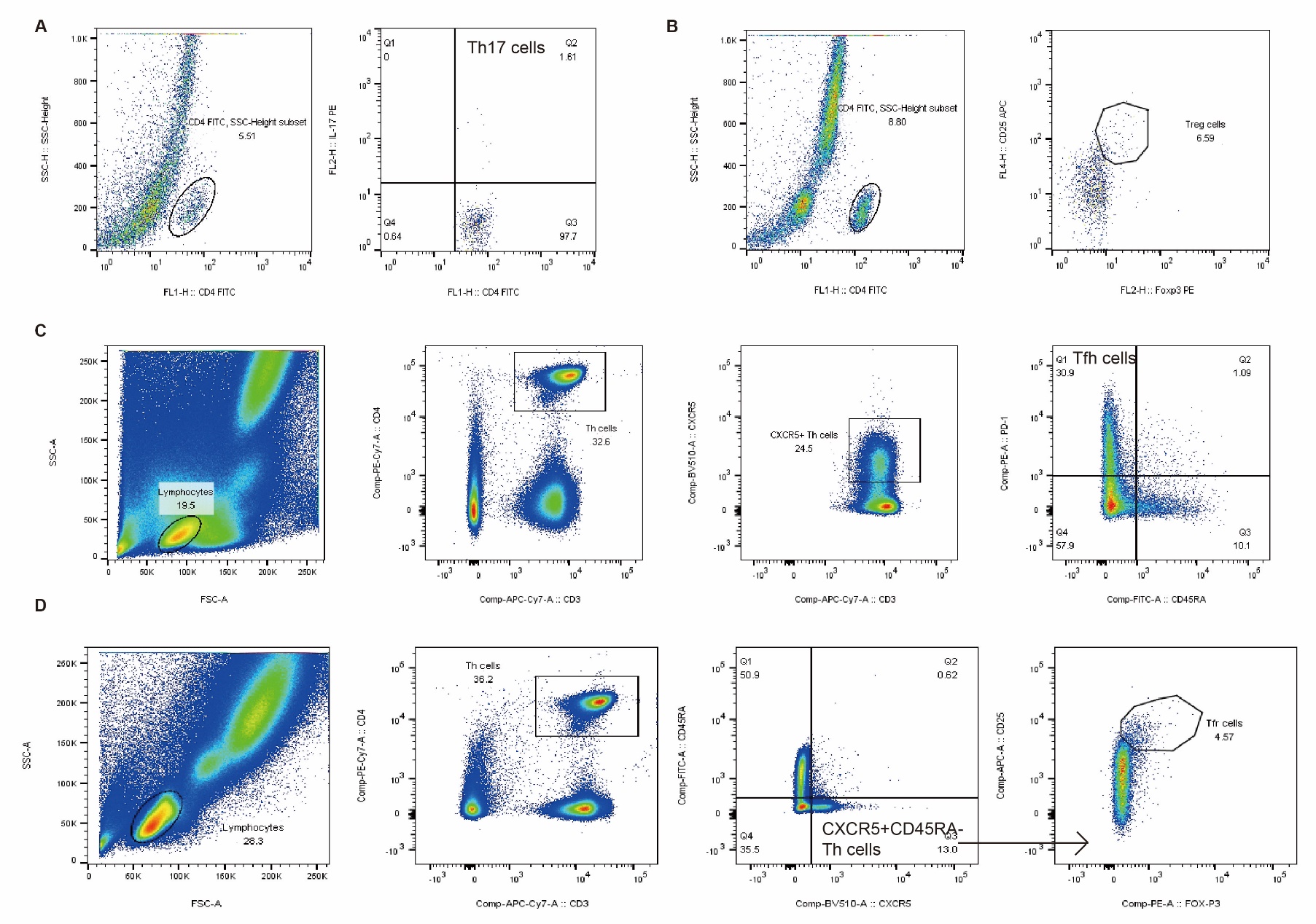


**Fig. S1.** The representative flow cytometry analysis of circulating Th17, Treg, Tfr and Tfh cells. (A)The circulating Th17 cells were identified as CD4+IL-17+T cells. (B)The circulating Treg cells were identified as CD4+CD25+FoxP3+T cells. (C) The circulating Tfh cells were identified as CD3+CD4+CXCR5+CD45RA-PD-1+ T cells. (D) The circulating Tfr cells were identified as CD3+CD4+ CXCR5+ CD45RA- CD25+ FoxP3+cells. (Th17: helper T 17 cells; Treg: regulatory T cells; Tfr: follicular regulatory T cells; Tfh: follicular helper T cells)


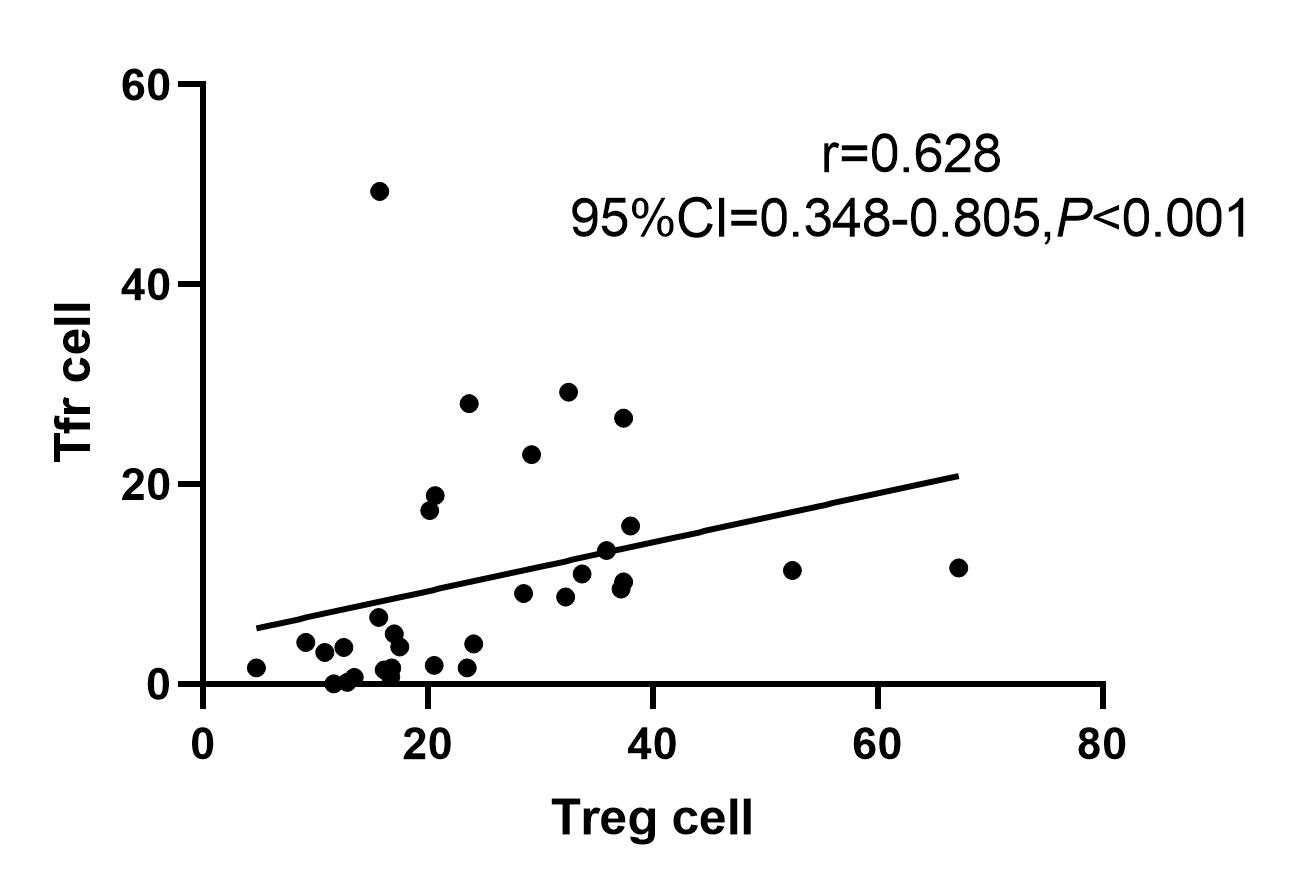


**Fig. S2.** The correlation between the number of Treg cells and Tfr cells in the new-onset RA patients. The two cells were related positively. (Treg: regulatory T cells; Tfr: follicular regulatory T cells; CI: confidence interval)


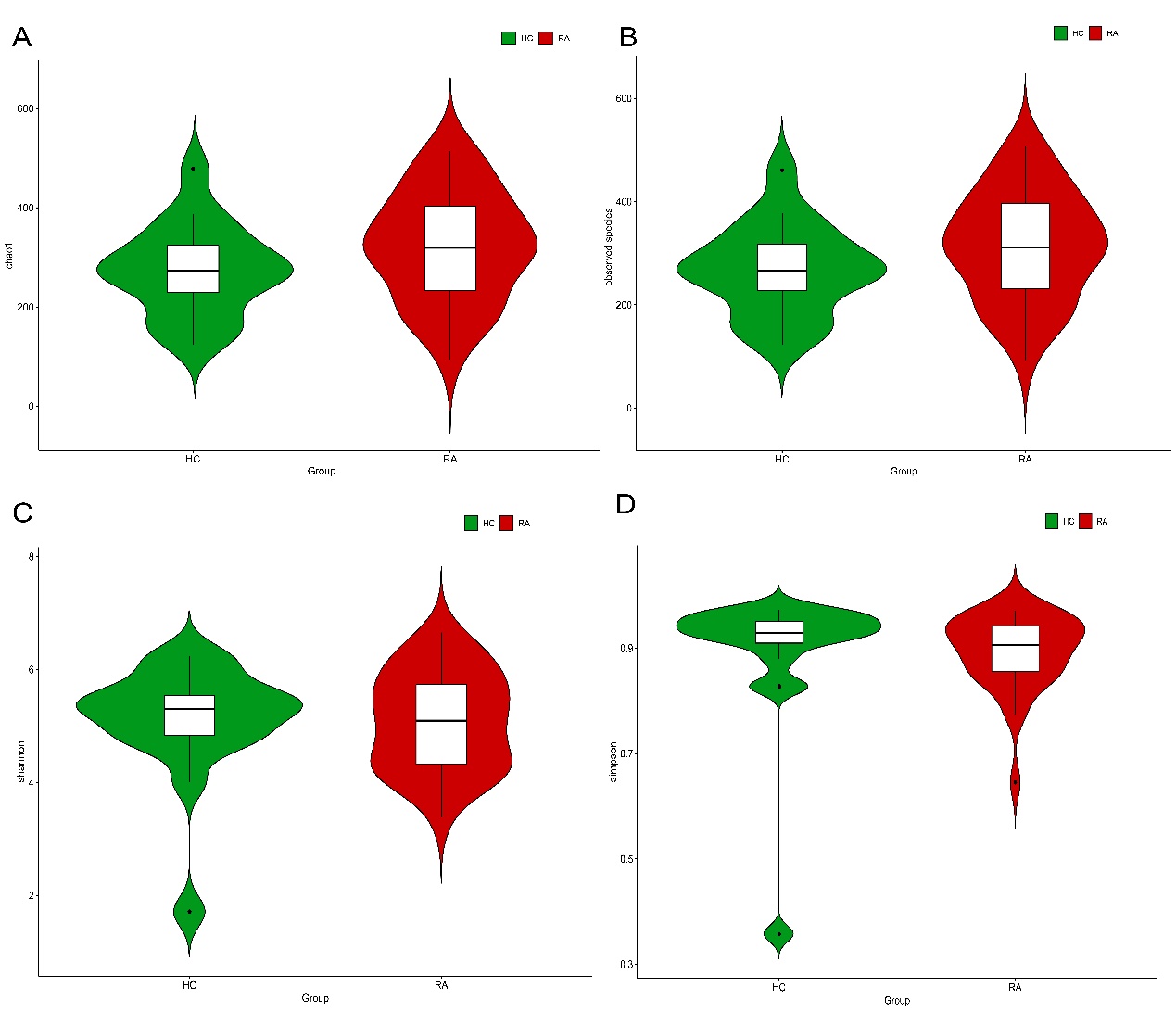


**Fig. S3.** The α-diversity analysis of new-onset RA patients and HCs. It was assessed by (A) Chao1, (B) Observed species, (C) Shannon and (D)Simpson indicators and showed that the species richness and evenness of gut microbiota in the new-onset RA patients were similar with that in HCs. (RA: rheumatoid arthritis; HCs: healthy controls)


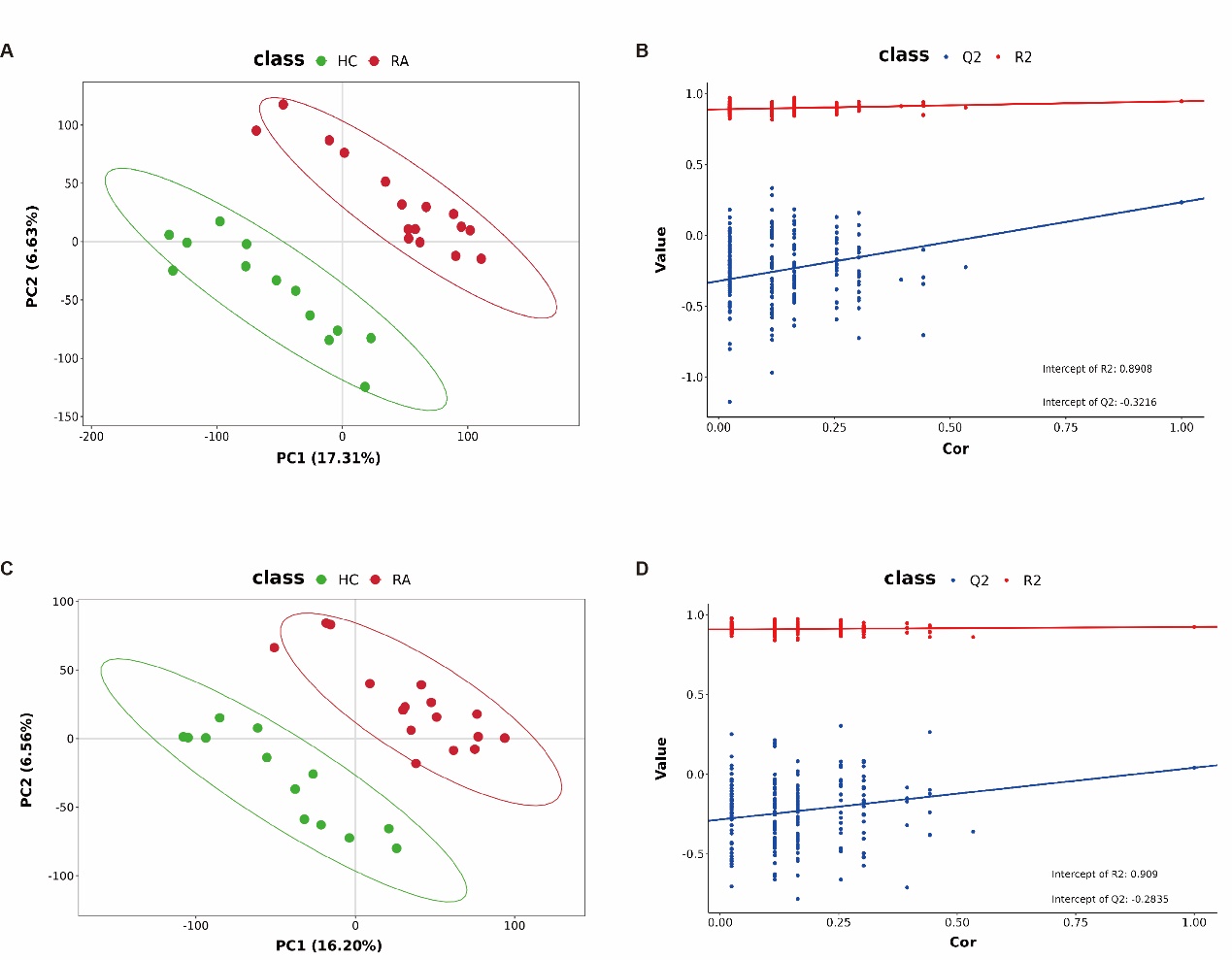


**Fig. S3.** Multivariate statistical analysis of fecal metabolite profiles between the RA and HCs. (A)The PLS-DA model showed that the fecal metabolites between the RA and HC were separated by differences in the positive ion mode. (B) The validation model of the PLS-DA model in the positive ion mode indicating that the PLS-DA model had a good ability of prediction and explanation without overfitting phenomenon (Intercept of R2 = 0.8908, Intercept of Q2 = -0.3216). (C) The PLS-DA model showed that the fecal metabolites between the RA and HC were separated by differences in the negative ion mode. (D) The validation model of the PLS-DA model in the negative ion mode indicating that the PLS-DA model had a good ability of prediction and explanation without overfitting phenomenon (Intercept of R2 = 0.909, Intercept of Q2 = -0.2835). (RA: rheumatoid arthritis; HCs: healthy controls; LDA: linear discriminant analysis; PLS-DA: projections to latent structures discriminant analysis)
